# Application of Optical Measurements to Assess Form Deviations of Cylindrical Parts Made Using FDM Additive Technology

**DOI:** 10.3390/s25185855

**Published:** 2025-09-19

**Authors:** Anna Bujarska, Paweł Zmarzły, Paweł Szczygieł

**Affiliations:** Faculty of Mechatronics and Mechanical Engineering, Kielce University of Technology, al. Tysiąclecia Państwa Polskiego 7, 25-314 Kielce, Poland; pzmarzly@tu.kielce.pl (P.Z.); pszczygiel@tu.kielce.pl (P.S.)

**Keywords:** fused deposition modeling, cylindricity, roundness deviations

## Abstract

Fused Deposition Modeling (FDM), also known as Fused Filament Fabrication (FFF), is a widely used additive manufacturing (AM) method for thermoplastic materials due to its low cost, accessibility, and ability to produce fully functional machine parts. Cylindrical components, common in mechanical devices, require precise dimensional and form accuracy to ensure long service life. To assess their quality, cylindricity deviation measurements are essential, as they reveal defects generated during the printing process. This study investigates the potential of optical scanning for measuring form deviations specifically cylindricity and roundness of ABS components manufactured via FDM. The influence of printing orientation (0°, 45°, 90°) on dimensional accuracy was examined using experimental models comprising three series of ten samples each, with identical process parameters except orientation. Measurements were performed using a Zeiss Prismo Navigator (Zeiss, Oberkochen, Germany) coordinate measuring machine and an ATOS II Triple Scan (GOM, Brunswick, Germany) optical scanner. Results indicate that print orientation significantly affects cylindricity deviation. The lowest deviations were achieved for specific orientations, offering guidelines for producing cylindrical surfaces of acceptable quality. The findings also show that optical scanners are not suitable for precise form deviation analysis in FDM-printed parts, confirming the higher accuracy of tactile coordinate measurement methods.

## 1. Introduction

The standard PN-EN ISO/ASTM 52900 [1] defines categories of additive manufacturing such as volumetric photopolymerization (VPP—VAT Photopolymerization), binder jetting (BJT), or the most popular material extrusion (MEX). The latter includes Fused Deposition Modeling (FDM), Fused Filament Fabrication (FFF), and Layer Plastic Deposition (LPD) technologies. The present study was conducted using the FDM technology.

FDM, commonly referred to as plastic 3D printing, is the most popular AM method. developed by Stratasys in the late 1980s, it is one of the most frequently used MEX technologies [2]. Its popularity is primarily due to its accessibility, low cost, and simple printing process, which quickly became available to users without prior experience in additive technologies. The FDM method involves building objects by layering material to generate three-dimensional elements [3]. This method involves the controlled extrusion of thermoplastic filaments through a heated nozzle. The construction material—a thermoplastic filament—is fed into the extruder head and heated to a specified temperature [4]. The printing process begins with the extrusion of molten filament through the nozzle, which deposits it line by line on the build platform. This process is repeated layer by layer until the entire object is produced [5]. Before applying the next layer to the previously printed layer, it is important that it undergoes a cooling and hardening process.

A distinctive feature of FDM is the need for support structures, built alongside the model or using a support material. These are necessary for overhangs or suspended in mid-air. While support enables the printing of geometrically complex shapes, their removal can be problematic [6,7].

Layer-by-layer production allows the creation of complex, composite, and hybrid structures with precision and control that cannot be achieved using traditional manufacturing methods [8]. The quality of the printed model depends on numerous printing parameters such as print angle, infill density, printing time, or layer thickness. The object is typically defined by a three-dimensional geometric model created using CAD (Computer-Aided Design) software. Three-dimensional printing software typically assumes that the geometry is represented as a set of triangles approximating the object’s surface. This industry-standard approach is implemented in the STL (Stereolithography/Standard Tessellation Language) file format [9]. STL simplifies the model by tessellating its surface into a mesh of triangles, each defined by vertex coordinates and a normal vector pointing outward. This principle translates complex geometries into a universal “triangle language” understood by 3D printers [2].

To reduce production costs and achieve the highest possible quality with minimal form errors, STL file parameters must be optimized. The authors of [10] focus on STL file analysis as the basis for 3D printing. Their study shows that errors in STL structure can result in incorrect geometry reproduction, affecting part quality and functionality.

In this article, attention is focused on cylindrical elements, which were selected for experimental research. Cylindrical components are found in almost every machine, and their performance and durability depend on precision manufacturing. However, the FDM process cannot produce a perfectly circular cross-section. Therefore, controlling form deviations in these elements is crucial.

Numerous studies have investigated the mechanical strength of printed parts [11,12], as well as the optimization of FDM process parameters for different materials. For example [13], analyzed infill patterns and nozzle temperature without emphasizing print orientation, demonstrating that appropriate optimization of 3D printing and post-processing parameters can significantly improve the tensile strength of PLA specimens. Using the Taguchi method, they identified key process parameters influencing mechanical strength—an important consideration for high-strength FDM applications.

The authors of [14] focused on how process parameters affect the geometric and dimensional accuracy of printed parts, investigating features such as cylindricity, perpendicularity, and surface roughness. They found no universal optimal settings, instead recommending process parameter customization for specific geometries. Similarly [11], studied PLA components, analyzing the influence of 3D printing parameters on mechanical properties and failure modes.

A comparison of cylindricity and roundness measurements conducted using a coordinate measuring machine (CMM) and a scanner [15] indicated that 3D scanning can be used for quality control only when high tolerances are acceptable. In contrast [16], observed that scanning enables measurement of entire object surfaces, including those with complex geometries, although it cannot measure internal surfaces (e.g., holes, grooves) and generally offers lower accuracy than CMM.

An analysis of the literature reveals a lack of studies focusing specifically on the influence of printing direction on cylindricity and roundness deviations of cylindrical elements. These deviations are critical in the manufacturing of cylindrical parts, making their investigation essential. In this work, the authors concentrated on cylindrical elements, measuring cylindricity and roundness deviations using both a CMM and an optical scanner. The obtained values were compared and analyzed.

## 2. Materials and Methods

### 2.1. Materials

The material used in the study was ABS (acrylonitrile-butadiene-styrene), commercially known as ABSplus P430 (Stratasys, Eden Prairie, MN, USA). This material is characterized by dimensional stability and wear resistance, making it suitable for both custom and mass production of mechanical components [17]. Table 1 presents selected mechanical properties.

The applied technology required the use of support structures. A soluble material, P400SR Soluble Support, was used for this purpose. The support material was removed using an SCA-1200 cleaning station (Stratasys, Eden Prairie, Minnesota). The cleaning process lasted approximately four hours.

### 2.2. Methods

**Figure 1:**
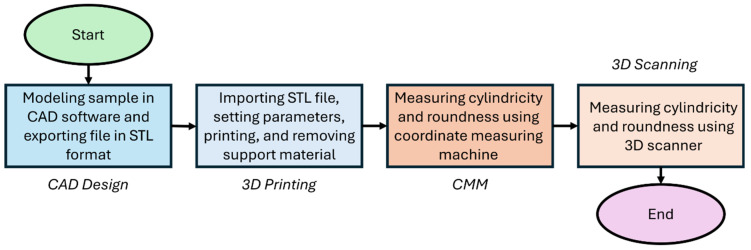
Flow chart of research procedure.

As part of the study, a sample model was created in CAD software, which was then saved in STL format. The model was produced using 3D printing technology in a quantity of 30 pieces, after which the support material was removed. The samples were then subjected to geometric measurements using a coordinate measuring machine (CMM) to determine cylindricity and roundness deviations. Following this, a 3D scanning process of the samples was conducted to obtain a mesh model, which was then aligned with the nominal model. Based on this, cylindricity and roundness analyses were performed to compare the results obtained by both methods (Figure 1).

### 2.3. Research Model

**Figure 2:**
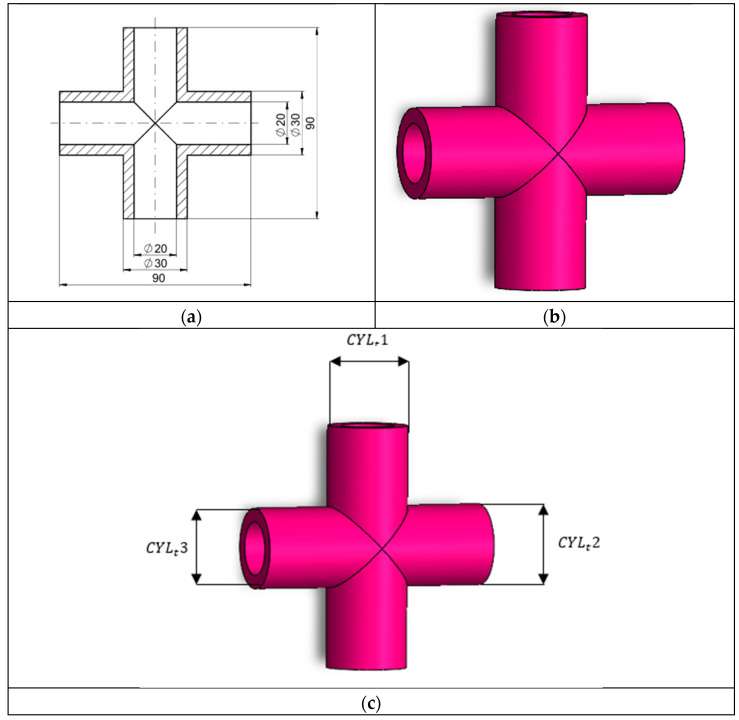
Sample: (**a**) dimensions, (**b**) 3D model, (**c**) designation of geometrical features.

The samples were designed using SOLIDWORKS 2023 software (Dassault Systèmes, Vélizy-Villacoublay, France). Since this program is one of the most widely used in industrial environments, its selection appears to be appropriate from a practical standpoint. Figure 2 shows the geometry of the printed sample. The part consists of two mirrored cylinders forming a component referred to as a quadripole. This element incorporates through-holes in its design. Due to significant aspects related to assembly, it is necessary to examine parameters describing both the dimensional and geometrical accuracy of external and internal surfaces. Owing to its design, the component can be applied in the construction of various machines, either as a connector between parts or as a pneumatic element. It may also be used in a wide range of hydraulic applications.

### 2.4. Three-Dimensional Printing Technology

The process of producing research samples used FDM technology classified in [1] as an additive manufacturing technology MEX. The Dimension SST 1200es (Stratasys, Eden Prairie, Minnesota) machine was employed. To build the three-dimensional element, this system extrudes ABS thermoplastic through a computer-controlled extrusion head, producing high-quality parts ready for immediate use.

**Figure 3:**
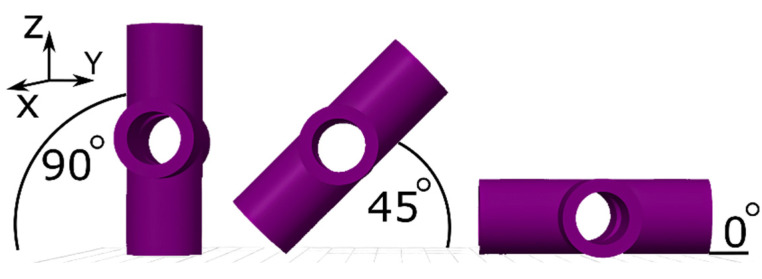
Arrangement of the samples on the 3D printer build platform.

In the applied printer, the extrusion head heats the material to its melting temperature and moves along the X and Y axes, while the build platform moves along the Z axis. The machine uses two print nozzles: the first for the model material and the second for the support material. It is equipped with a built platform of 254 mm × 254 mm × 305 mm, and the chamber reaches a temperature of 200 °C. The technological parameters used for the printing were a layer height of 0.254 mm and a high-density infill type for all samples. Figure 3 shows the arrangement of the samples on the printer’s build platform. A total of three samples (in each of the three orientations) were produced in a single print job, and ten print jobs were carried out, resulting in 30 samples in total.

### 2.5. Measurement of Cylindricity and Roundness Deviation

According to the definition provided in PN-EN ISO 1101:2017-05 [19] (Geometrical Product Specifications (GPS)–Geometrical Elements–Part 1: Basic terms and definitions), the cylindricity deviation (∆C) is the maximum distance of the points on the actual surface from the associated cylinder surface, within the boundaries of a partial area. The associated cylinder is the cylinder with the smallest diameter that encloses the shaft surface or the largest diameter cylinder that can be inscribed into the surface of a given hole.

The least squares method allows for the simplest determination of the so-called reference cylinder. With respect to this reference cylinder, the parameter denoted as CYLt is defined; it represents the sum of the largest absolute positive value and the largest absolute negative value of the local cylindricity deviations measured in relation to the associated cylinder. This parameter is expressed by Equation (1).

(1) CYLt=CYLp+CYLv

where CYL_p_ is the value of the largest positive local cylindricity deviation measured with respect to the reference cylinder determined using the least squares method, and CYLv is the absolute value of the largest negative local cylindricity deviation with respect to the same reference cylinder, also determined using the least squares method.

Cylindricity deviation can be analyzed using specialized measurement systems based on the method of measuring radial variation. Measurements performed with such systems are considered reference-grade. However, these systems are expensive and are typically used only in laboratory conditions. Consequently, current industrial practice relies on more versatile measurement solutions, such as coordinate measurement techniques or optical systems (optical scanners).

The roundness deviation (RONt) is defined as the difference between the maximum and minimum deviation of the profile from the reference circle, or equivalently, as the sum of the positive deviation RONp and the negative deviation RONv relative to the reference circle.

*RONt* = *RONp* + *RON_V_* (2)

where RONp is the value of the highest peak in the roundness profile, and RONv is the largest depth in the roundness profile.

A key factor in measuring deviations is the measurement strategy and the number of measurement points. The number of points should be strictly defined prior to the measurement process. The measurement strategy, a term inherently linked with the path of the probe, refers to the so-called scanning trajectory [20].

Coordinate measuring techniques differ from classical metrology in their measurement strategy. They rely on computer-processed discrete measurement data and allow the determination of the dimensions of spatially complex machine parts with relatively high accuracy, often within cycle times compatible with production [21]. A coordinate measuring machine (CMM) is a device in which the measuring components move along defined coordinates, with at least one axis providing movement. The directions are determined by the X, Y, and Z axes of the Cartesian coordinate system, which define the machine’s reference frame. The relative movement between the probe system and the measured point on the component is realized through linear axes. Contact with the part is established by probing tips of various geometries—most commonly spherical, but also conical, disk-shaped, or cylindrical. The geometry of the probe tip must be specified before measurement, during a calibration process involving the measurement of a reference artifact so that its size and position can be compensated in all subsequent measurements.

The advantages of optical measuring devices include short setup times, ease of operation, and generally lower cost compared to coordinate measuring machines. In addition, optical measurement is non-contact, allowing the analysis of elastic components, particularly those made of soft polymers produced by 3D printing. Optical measurements can be partially automated; although automation is also possible with CMMs, the technical limitations of tactile measurement may make it less cost-effective than optical systems. It should be noted, however, that contact measurement is generally more accurate and can be applied to a wide range of geometries, not limited to cylindrical shapes [22].

For the measurements, a Zeiss Prismo Navigator coordinate measuring machine (Zeiss, Oberkochen, Germany) with Zeiss Calypso 2015 (Zeiss, Oberkochen, Germany) software and an ATOS II Triple Scan optical scanner were used. The Zeiss Prismo Navigator was equipped with a Zeiss VAST Gold scanning probe head, which, thanks to the Navigator system, allows scanning at variable speeds, including high speeds up to 300 mm/s. The use of active scanning heads improves the accuracy of form deviation measurements. Table 2 presents the specifications of the selected measuring machine.

A probe with a 3 mm diameter ruby stylus tip was used. The number of measurement points for both roundness and cylindricity (measured in three cross-sections) was 1800 per diameter. The scanning speed was set to 15 mm/s. A measurement strategy known as the cross-sectional strategy was applied, in which the roundness profile is measured repeatedly along the length of the part, with equal spacing between the profiles. In this study, measurements were taken in five cross-sections. The expanded measurement uncertainty of the coordinate measuring machine (CMM) was 2 µm at a confidence level of approximately 95%, with a coverage factor of k = 2. A Gaussian filter 1–50 UPR was applied. Environmental conditions maintain strict temperature control within a ±0.5 °C range. The CMM was equipped with an additional temperature compensation sensor.

Measurements were carried out under controlled conditions at an ambient temperature of 20 °C and relative humidity of 55%, by a single operator.

**Figure 4:**
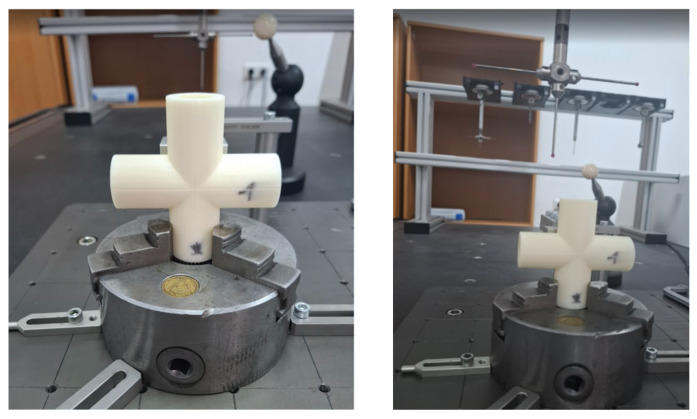
Mounting the sample on the coordinate measuring machine.

Figure 4 shows the mounting of the research sample on the coordinate measuring machine. The mounting used for all samples.

In the next stage of the study, according to the adopted procedure (Figure 2), a 3D inspection was performed using an ATOS II Triple Scan optical scanner with the GOM Inspect Pro 2021 software (GOM Co., Brunswick, Germany). The scanner head was positioned automatically. After measurement, the scanner head or the scanned item was moved to measure areas that were not scanned in the previous positioning. The entire measurement was automatically transformed to a common coordinate system and generated a cloud of 3D points. Table 3 shows the technical specifications of the 3D scanner.

The ATOS II Triple Scan 3D scanner operates using the structured-light method with blue-light fringe projection and a dual-camera triangulation setup. Spatial point data is captured by computing the intersection between the projected structured-light fringe pattern and each camera pixel ray. Because the system uses two cameras simultaneously (over-determination), measurement accuracy and process reliability are enhanced. Captured deformation patterns are processed by dedicated software to produce a high-resolution height map and a dense three-dimensional point cloud.

**Figure 5:**
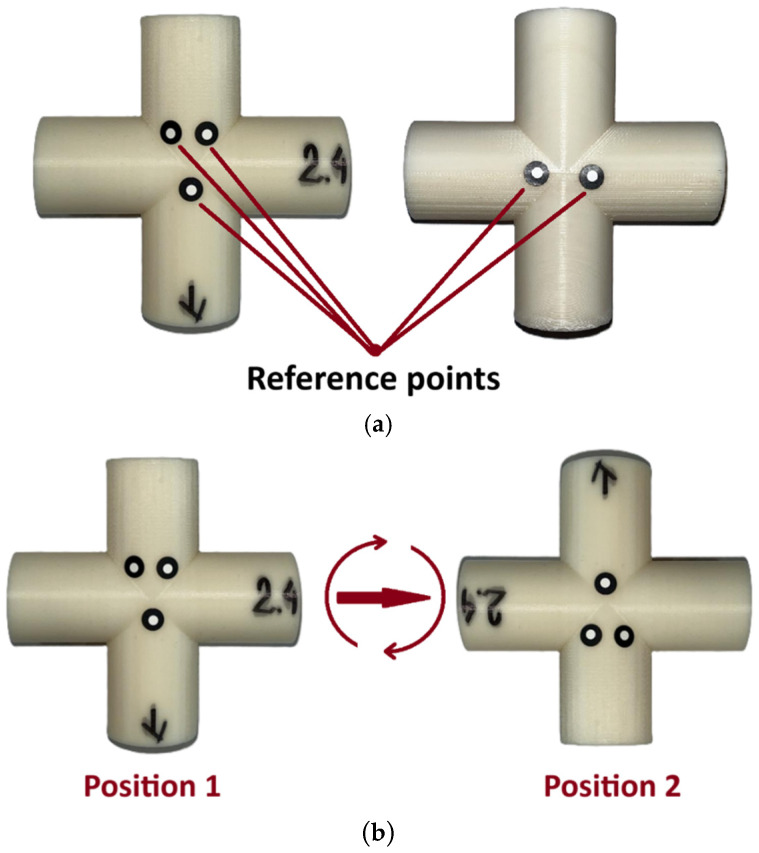
Process of preparing the sample for 3D scanning (**a**) applying reference points, (**b**) scanning positions.

Before starting the 3D scanning process, five reference points (so-called markers) were applied to each sample. These markers enabled precise alignment of point clouds obtained during digitization in different positions (Figure 5a). Each sample was scanned in two positions (Figure 5b), which allowed for capturing all parts of the external surfaces.

To fully capture the geometry, 18 scanning steps were defined, which, with one complete rotation of the scanner table around its axis, resulted in scans taken every 20°. This approach allowed for high point cloud density and good-quality geometric data.

**Figure 6:**
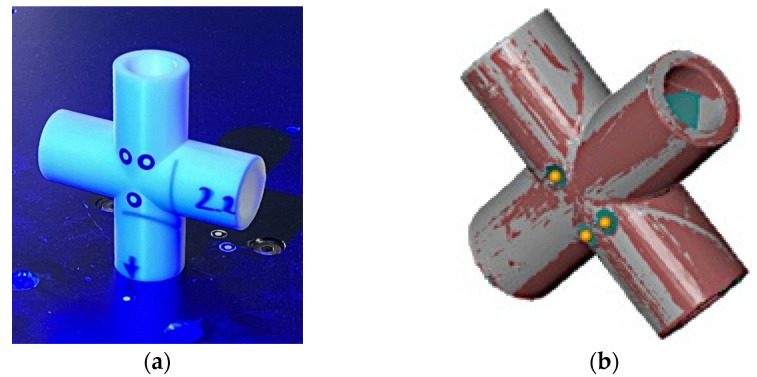
Three-Dimensional scanning process (**a**) positioning of the sample in the first position, (**b**) transformation of the point clouds.

The use of an anti-reflective coating was omitted, as the surface properties of the samples allowed for effective scanning with the scanner’s automatic light intensity adjustment (Figure 6a). This made it possible to preserve the natural surface characteristics without introducing additional layers that could affect the measurement results.

After performing two scans for each sample, the resulting point clouds were merged using the previously applied markers (Figure 6b). The measurement software automatically aligned the data sets and calculated the alignment deviation, which for sample 2.4 was 0.03 mm. This value represents the average spatial difference between corresponding points in the two clouds after alignment. In other words, after the transformation, the average shift in points between the clouds was 0.03 mm. This deviation falls within the accuracy range typical for this type of measurement and was recognized by the software algorithm as a high-quality match, enabling further geometric analysis. The average alignment deviation across all samples was 0.024 mm.

**Figure 7:**
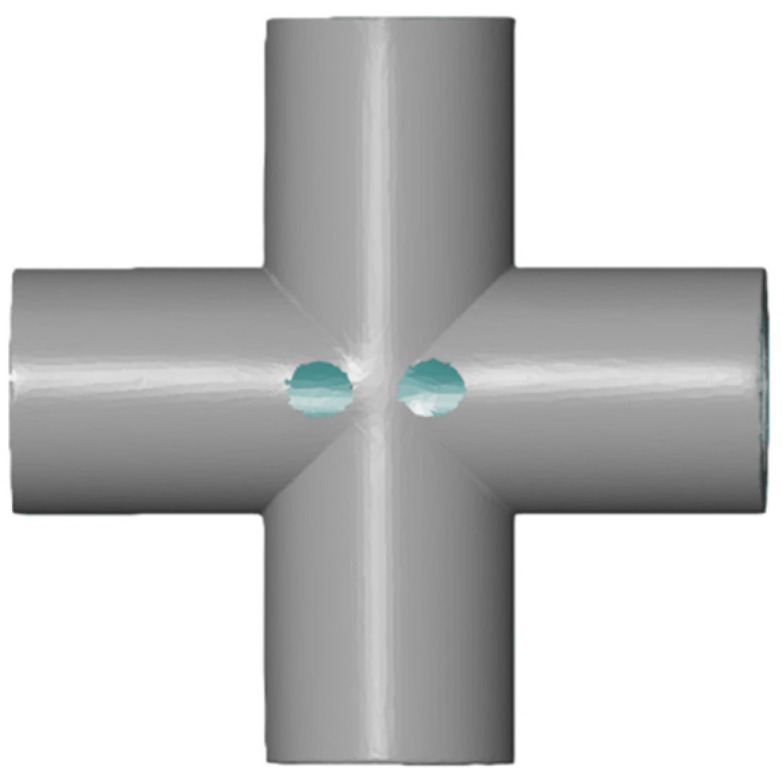
Sample after the polygonization process.

Next, the process of polygonization was carried out, which involved converting the point cloud into a surface model in the form of a triangular mesh. During this process, holes left by the reference markers and gaps in hard-to-reach areas (e.g., the interior of holes) were intentionally left unfilled, in order to avoid introducing artificial data that could distort the actual geometric condition of the samples and affect the analysis results, as shown in Figure 7.

As a result, the cylindricity and roundness analysis was conducted exclusively on the external surfaces of the samples. Similarly to the measurements performed using a coordinate measuring machine (CMM), three selected cylindrical surfaces were analyzed, maintaining their order and designations consistent with the earlier stages of the study.

After completing the 3D scanning process and obtaining the actual surface model in the form of a triangular mesh, a geometric analysis was carried out by comparing the real model to the nominal (CAD) model. This comparison enables the identification of form deviations, such as cylindricity and roundness, relative to the designed (ideal) geometry.

**Figure 8:**
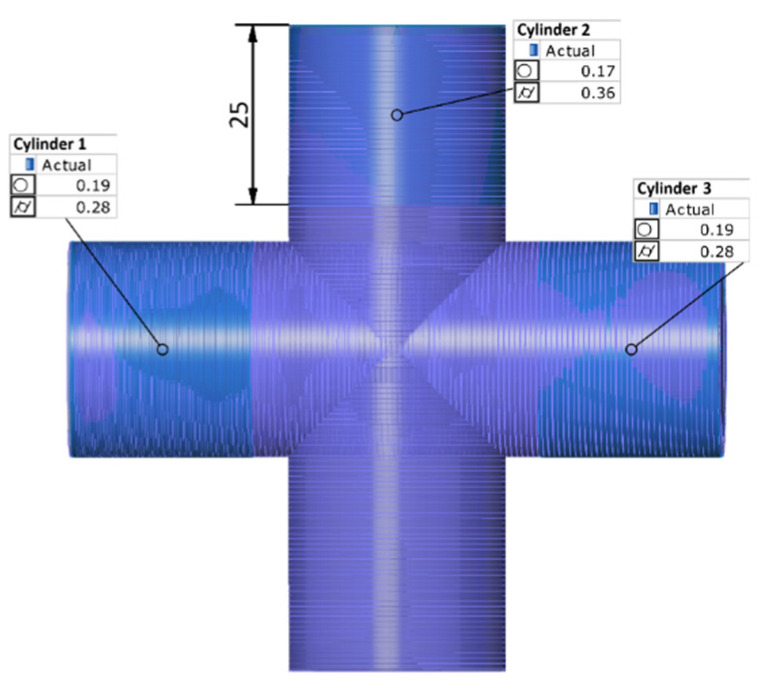
Three-Dimensional scanning result.

In the first step, the scan was aligned with the nominal model using the best-fit function. During this step, the software recorded the base deviation (the average deviation for all samples was 0.06 mm). In the next stage, within the GOM Inspect software, cylindrical surfaces were defined by selecting the appropriate areas of the mesh that were to undergo geometric analysis (with the cylinder height set to 25 mm). Based on the selected mesh segments, the software automatically fitted the ideal cylinder using the Best-fit Gauss method. Cylindricity and roundness deviations were determined, and the results were presented numerically (Figure 8).

The optical scanner captured deviations in the structured light fringes relative to two cameras, generating a point cloud. During a single measurement series, the part was scanned multiple times while the scanner table rotated (a full rotation of the table was divided into 18 steps), and the resulting point clouds were automatically merged into a single data set. The part was then repositioned to scan areas that were not visible in the previous series; the newly acquired point clouds were transformed and merged with the earlier ones, which sometimes led to distortions in the reproduced geometry.

In areas where the camera failed to register points, the software filled the gaps during the polygonization process using flat surfaces, which affected the shape and the values of deviations. Fitting the resulting 3D model to the CAD model also introduced additional deviations.

In studies involving the assessment of roundness and cylindricity, the limitations of the optical method such as light reflections, difficulties in capturing the gaps between FDM layers (staircase effect), and the accumulation of errors during the alignment and merging of multiple scans may affect the accuracy of the measurements when compared to those obtained using a coordinate measuring machine (CMM).

## 3. Results and Discussion

Table 4 presents the results of the measurements of roundness deviation measured using CMM and a scanner.

Table 5 shows the results of measuring the cylindricity deviation using CMM and a scanner.

A statistical analysis was performed for individual printing angles of the samples. The mean and standard deviation were calculated. An analysis of the percentage differences in individual measurements was conducted, as shown in Table 6.

Analyzing the results presented in Table 6, it can be concluded that the highest average roundness deviation values were obtained for the printing direction Pd = 0°, while the smallest values were observed for Pd = 45° (both for contact and optical measurements). It should be noted that the most noticeable differences between the measurement methods were observed for the details printed at an angle of Pd = 0°.

When evaluating the roundness deviation measurement methods, it can be concluded that in most of the analyzed cases, the roundness deviations measured using the optical scanner exceeded those obtained from contact measurements with the CMM. Furthermore, it can be stated that contact measurements exhibited higher repeatability compared to optical measurements. For the printing direction Pd = 45°, noticeable differences were observed between the roundness deviation values measured using both contact and optical methods. Therefore, it can be concluded that for smaller roundness deviations, contact measurements performed with the CMM are recommended.

**Figure 9:**
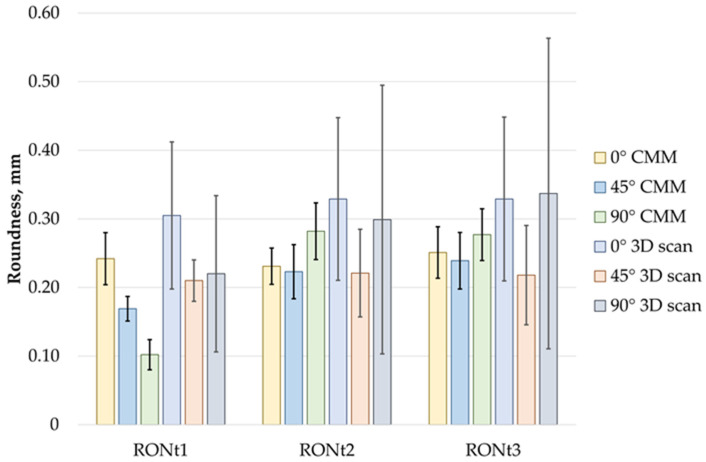
Comparison of roundness measurement results.

Similarly, when analyzing the measured cylindricity deviations, similar conclusions can be drawn as for the roundness measurements. Here, the smallest cylindricity deviation values were also observed for the printing direction Pd = 45°, while the largest values were found for Pd = 0°. As with roundness, the cylindricity values obtained from the scanner exceeded those measured with the CMM. A comparison of the results is shown in Figure 9.

Figure 9 presents a comparison of roundness measurement results for three analyzed cases: RONt1, RONt2, and RONt3, obtained using two measurement methods: the coordinate measuring machine (CMM) and the 3D scanner. The average roundness values range from 0.10 mm to 0.34 mm.

In the 0° orientation, the average values obtained with the scanner were higher than the CMM results by 29% for RONt1, 43% for RONt2, and 32% for RONt3, with the standard deviation being more than twice as large. In the 45° orientation, the average values were closer, but the standard deviations for the scanner were still two to three times larger than those for the CMM measurements, indicating lower repeatability of the scanner method. The largest discrepancies were observed in the 90° orientation, where for RONt1, the scanner result was 120% higher than the CMM result, and the standard deviation increased more than fivefold. Similarly, for RONt2 and RONt3, the scanner’s standard deviations were approximately five times greater than those for the CMM, despite the smaller differences in average values.

**Figure 10:**
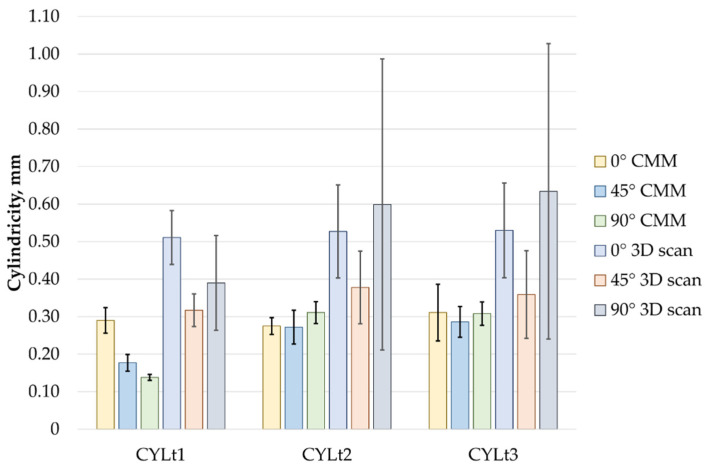
Comparison of the measurement results of cylindricity.

Figure 10 presents a comparison of cylindricity measurement results for three cases: CYLt1, CYLt2, and CYLt3, using two methods: the coordinate measuring machine (CMM) and 3D scanning, performed in three orientations: 0°, 45°, and 90°. The average cylindricity values range from 0.14 mm to 0.63 mm.

In the 0° orientation, the cylindricity values measured using the scanner were higher than those obtained with the CMM. For CYLt1, the scanner result was 76% higher, while for CYLt2 and CYLt3, it was 89% and 71% higher, respectively. In each case, the standard deviation of the results obtained with the scanning method was also higher, ranging from about 2 to over 4 times greater.

In the 45° printing orientation, for CYLt1, the scanner result was 78% higher, for CYLt2 it was 41%, and for CYLt3 it was 24%. Standard deviations for the scanner were 2 to 3 times greater than those for the CMM.

The largest discrepancies occurred in the 90° orientation, where the 3D scanning method significantly overestimated the cylindricity values. For CYLt1, the difference from the CMM result was 179%, for CYLt2 it was 94%, and for CYLt3 it was 103%. Moreover, the standard deviations for the 3D scanning measurements for CYLt2 and CYLt3 were more than 13 times larger than those from the CMM measurements.

In summary, the elevated roundness and cylindricity deviations measured with optical methods may stem from the measurement principle itself. In the case of contact measurements with a CMM, a scanning probe with a ruby ball of appropriate diameter is used. The contact probe acts as a mechanical-geometry filter. Additionally, some contaminants on the measured surface can be removed by contact with the ruby ball. In contrast, optical measurements scan the entire surface of the printed element. If contaminants are present, the scanner may interpret them as part of the measured geometry, ultimately leading to an overestimation of the measured deviations.

To conduct a more detailed analysis of the obtained results, graphical representations in the form of charts were used.

## 4. Summary and Conclusions

The Fused Deposition Modeling (FDM) technology, due to its popularity, is increasingly used for manufacturing fully functional parts that do not require further processing, referred to as post-processing. This applies especially to cylindrical components. In such cases, measuring only dimensional deviations proves insufficient and does not provide complete information about the quality of the manufactured parts. Therefore, it becomes necessary to analyze roundness and cylindricity deviations, which help detect defects arising both from the printing process and the applied technological parameters.

Currently, in most cases, such deviations are measured using specialized instruments or coordinate measuring machines (CMM) that rely on contact measurements. While these methods are precise, they are time-consuming and costly. As a result, non-contact measurements using optical scanners have become an alternative. Based on the research presented in this article, the following conclusions were drawn:

- Measurements obtained using the CMM were characterized by lower values compared to those obtained with 3D scanning.
- Analyzing roundness and cylindricity deviations of cylindrical components made using FDM technology in three different printing orientations (0°, 45°, 90°) clearly shows a dependence of geometric quality on the printing orientation. The largest deviations in both roundness and cylindricity were observed for the 90° printing direction, likely due to deformations resulting from the layer-by-layer material deposition and settling of successive layers.
- Roundness and cylindricity measurements of cylindrical components produced by FDM technology clearly indicate that surface 3 exhibited the highest values for roundness and cylindricity deviations, regardless of the printing direction (0°, 45°, 90°) and the measurement method (CMM and 3D scanner). The smallest deviations were recorded for Feature 1, with minor exceptions that did not significantly impact the overall trend.
- Summarizing the results, it can be concluded that contact measurements with a CMM provide greater accuracy and repeatability than 3D scanning, which, in turn, offers faster and more comprehensive surface quality analysis, albeit with noticeably overestimated deviation values.
- The choice of measurement method should depend on the quality requirements for the intended application of the manufactured part. For high-accuracy requirements, contact measurements are recommended, while for parts with larger tolerances, optical measurements can be an effective alternative.

As a direction for future research, the Authors plan to investigate the use of optical scanners to assess other geometric features. Additionally, models will be produced using other additive manufacturing technologies (PJM, SLS).

Considering the obtained results, it is recommended that for low-dimensional accuracy (large tolerances) and time-saving, 3D scanning should be selected. Conversely, for precise measurements where accuracy is crucial, CMM should be used.

## Figures and Tables

**Table 1:** Selected mechanical properties [18].

Mechanical Properties	Value	Unit	Test Standard
Tensile Strength, Ultimate (Type 1, 0.125″, 0.2″/min)	33	MPa	ASTM D638
Tensile Strength, Yield (Type 1, 0.125″, 0.2″/min)	31	MPa	ASTM D638
Tensile Modulus (Type 1, 0.125″, 0.2″/min)	2200	MPa	ASTM D638
Tensile Elongation at Break (Type 1, 0.125″, 0.2″/min)	6	%	ASTM D638
Tensile Elongation at Yield (Type 1, 0.125″, 0.2″/min)	2	%	ASTM D638
IZOD Impact, notched (Method A, 23 °C)	106	J/m	ASTM D256

**Table 2:** Coordinate machine parameters.

Parameter	Value
Measurement range	X = 900 mm; Y = 1200 mm, Z = 700 mm
Max. permissible error/spindle error	0.9 + L/350 µm

**Table 3:** Technical specification of the 3D scanner.

Camera Pixels	Measuring Area (mm^2^)	Point Spacing (mm)	Recording of Measured Points	Working Distance (mm)	Operating Temperature (°C)	Rotary Table	Permissible Measurement Limit Error (mm)
2 × 5,000,000	38 × 29–2000 × 1500	0.02–0.79	up to 1,400,000	490–2000	5–40 (non-condensing)	Yes	±0.01

**Table 4:** Results of roundness deviation measurements.

		RONt CMM			RONt 3D Scan			Percentage Difference		
	**Sample no.**	**RONt 1 mm**	**RONt2 mm**	**RONt3 mm**	**RONt1 mm**	**RONt2 mm**	**RONt3 mm**	**RONt1 %**	**RONt2 %**	**RONt3 %**
**print direction 0°**										
	**1_1**	0.29	0.24	0.28	0.25	0.29	0.29	13.79	17.24	3.45
	**1_2**	0.24	0.24	0.24	0.4	0.25	0.25	40.00	4.00	4.00
	**1_3**	0.17	0.27	0.24	0.16	0.29	0.29	5.88	6.90	17.24
	**1_4**	0.23	0.26	0.34	0.18	0.26	0.26	21.74	0.00	23.53
	**1_5**	0.22	0.19	0.24	0.5	0.66	0.66	56.00	71.21	63.64
	**1_6**	0.3	0.22	0.26	0.31	0.32	0.33	3.23	31.25	21.21
	**1_7**	0.25	0.19	0.23	0.33	0.29	0.27	24.24	34.48	14.81
	**1_8**	0.27	0.22	0.25	0.26	0.32	0.33	3.70	31.25	24.24
	**1_9**	0.22	0.24	0.20	0.25	0.30	0.30	12.00	20.00	33.33
	**1_10**	0.23	0.24	0.23	0.41	0.31	0.31	43.90	22.58	25.81
**print direction 45°**										
	**2_1**	0.18	0.22	0.23	0.19	0.28	0.29	5.26	21.43	20.69
	**2_2**	0.16	0.23	0.27	0.26	0.18	0.19	38.46	21.74	29.63
	**2_3**	0.17	0.21	0.23	0.18	0.22	0.21	5.56	4.55	8.70
	**2_4**	0.19	0.13	0.13	0.17	0.19	0.19	10.53	31.58	31.58
	**2_5**	0.2	0.29	0.24	0.18	0.18	0.18	10.00	37.93	25.00
	**2_6**	0.15	0.24	0.27	0.24	0.19	0.19	37.50	20.83	29.63
	**2_7**	0.16	0.22	0.26	0.22	0.38	0.38	27.27	42.11	31.58
	**2_8**	0.17	0.24	0.24	0.24	0.22	0.23	29.17	8.33	4.17
	**2_9**	0.14	0.22	0.26	0.21	0.19	0.11	33.33	13.64	57.69
	**2_10**	0.17	0.23	0.26	0.21	0.18	0.21	19.05	21.74	19.23
**print direction 90°**										
	**3_1**	0.1	0.28	0.31	0.17	0.84	0.94	41.18	66.67	67.02
	**3_2**	0.11	0.22	0.21	0.19	0.24	0.45	42.11	8.33	53.33
	**3_3**	0.16	0.26	0.26	0.18	0.21	0.25	11.11	19.23	3.85
	**3_4**	0.1	0.3	0.28	0.17	0.2	0.2	41.18	33.33	28.57
	**3_5**	0.1	0.26	0.29	0.18	0.2	0.2	44.44	23.08	31.03
	**3_6**	0.08	0.27	0.22	0.18	0.26	0.26	55.56	3.70	15.38
	**3_7**	0.1	0.3	0.31	0.16	0.24	0.25	37.50	20.00	19.35
	**3_8**	0.09	0.28	0.3	0.54	0.36	0.37	83.33	22.22	18.92
	**3_9**	0.09	0.27	0.32	0.22	0.21	0.22	59.09	22.22	31.25
	**3_10**	0.09	0.38	0.27	0.21	0.23	0.23	57.14	39.47	14.81

**Table 5:** Results of cylindricity deviation measurements.

		CYLt, CMM			CYTt 3D Scan			Percentage Difference		
	**Sample no.**	**CYLt1 mm**	**CYLt2 mm**	**CYLt3 mm**	**CYLt1 mm**	**CYLt2 mm**	**CYLt3 mm**	**CYLt1 %**	**CYLt2 %**	**CYLt3 %**
**print direction 0°**										
	**1_1**	0.32	0.28	0.31	0.51	0.47	0.47	37.25	40.43	34.04
	**1_2**	0.30	0.29	0.29	0.59	0.46	0.46	49.15	36.96	36.96
	**1_3**	0.23	0.29	0.27	0.37	0.45	0.45	37.84	35.56	40.00
	**1_4**	0.30	0.31	0.51	0.46	0.41	0.41	34.78	24.39	19.61
	**1_5**	0.26	0.23	0.28	0.64	0.85	0.85	59.38	72.94	67.06
	**1_6**	0.34	0.28	0.32	0.51	0.49	0.51	33.33	42.86	37.25
	**1_7**	0.30	0.25	0.34	0.52	0.49	0.50	42.31	48.98	32.00
	**1_8**	0.32	0.27	0.28	0.52	0.52	0.52	38.46	48.08	46.15
	**1_9**	0.27	0.27	0.24	0.51	0.54	0.50	47.06	50.00	52.00
	**1_10**	0.26	0.28	0.27	0.48	0.59	0.63	45.83	52.54	57.14
**print direction 45°**										
	**2_1**	0.19	0.27	0.27	0.36	0.39	0.39	47.22	30.77	30.77
	**2_2**	0.20	0.26	0.28	0.30	0.28	0.29	33.33	7.14	3.45
	**2_3**	0.17	0.26	0.27	0.26	0.30	0.30	34.62	13.33	10.00
	**2_4**	0.22	0.16	0.20	0.36	0.28	0.28	38.89	42.86	28.57
	**2_5**	0.18	0.26	0.27	0.25	0.31	0.31	28.00	16.13	12.90
	**2_6**	0.16	0.30	0.30	0.30	0.50	0.49	46.67	40.00	38.78
	**2_7**	0.14	0.28	0.31	0.36	0.46	0.46	61.11	39.13	32.61
	**2_8**	0.17	0.31	0.36	0.37	0.52	0.47	54.05	40.38	23.40
	**2_9**	0.17	0.31	0.29	0.30	0.45	0.13	43.33	31.11	55.17
	**2_10**	0.17	0.31	0.31	0.31	0.29	0.47	45.16	6.45	34.04
**print direction 90°**										
	**3_1**	0.14	0.33	0.33	0.47	1.67	1.67	70.21	80.24	80.24
	**3_2**	0.15	0.25	0.23	0.31	0.55	0.55	51.61	54.55	58.18
	**3_3**	0.13	0.29	0.30	0.31	0.44	0.84	58.06	34.09	64.29
	**3_4**	0.14	0.30	0.33	0.30	0.38	0.38	53.33	21.05	13.16
	**3_5**	0.15	0.33	0.30	0.31	0.37	0.37	51.61	10.81	18.92
	**3_6**	0.14	0.30	0.31	0.32	0.47	0.45	56.25	36.17	31.11
	**3_7**	0.13	0.31	0.33	0.31	0.51	0.53	58.06	39.22	37.74
	**3_8**	0.13	0.32	0.30	0.67	0.70	0.70	80.60	54.29	57.14
	**3_9**	0.14	0.32	0.31	0.37	0.43	0.39	62.16	25.58	20.51
	**3_10**	0.13	0.36	0.34	0.53	0.47	0.46	75.47	23.40	26.09

**Table 6:** Mean values and standard deviation of roundness and cylindricity measurements.

Print Direction 0°	CMM		Scanner		Print Direction 0°	CMM		Scanner	
	$\bar{x}$, mm	SD, mm	$\bar{x}$, mm	SD, mm		$\bar{x}$, mm	SD, mm	$\bar{x}$, mm	SD, mm
**RONt1**	0.24	0.04	0.31	0.11	**CYLt1**	0.29	0.03	0.51	0.07
**RONt2**	0.23	0.03	0.33	0.12	**CYLt2**	0.28	0.02	0.53	0.12
**RONt3**	0.25	0.04	0.33	0.12	**CYLt3**	0.31	0.08	0.53	0.13
**print direction 45°**					**print direction 45°**				
**RONt1**	0.17	0.02	0.21	0.03	**CYLt1**	0.18	0.02	0.32	0.04
**RONt2**	0.22	0.04	0.22	0.06	**CYLt2**	0.27	0.04	0.38	0.10
**RONt3**	0.24	0.04	0.22	0.07	**CYLt3**	0.29	0.04	0.36	0.12
**print direction 90°**					**print direction 90°**				
**RONt1**	0.10	0.02	0.22	0.11	**CYLt1**	0.14	0.01	0.39	0.13
**RONt2**	0.28	0.04	0.30	0.20	**CYLt2**	0.31	0.03	0.60	0.39
**RONt3**	0.28	0.04	0.34	0.23	**CYLt3**	0.31	0.03	0.63	0.39

## Data Availability

Data are contained within the article.
